# A multicenter point-prevalence study: antimicrobial prescription frequencies in hospitalized patients in turkey

**DOI:** 10.1186/1476-0711-4-16

**Published:** 2005-10-03

**Authors:** Gaye Usluer, Ilhan Ozgunes, Hakan Leblebicioglu, the Turkish Antibiotic Utilization Study Group

**Affiliations:** 1 Osmangazi University, Faculty of Medicine, Department of Infectious Diseases, Eskisehir-Turkey; 2 Ondokuz Mayis University, Faculty of Medicine, Department of Infectious Diseases, Samsun-Turkey

**Keywords:** antimicrobial use, appropriate antimicrobial use, cost

## Abstract

**Background:**

Accurate information about prescribing patterns in hospitals is valuable in improving the quality of antimicrobial prescriptions.

**Methods:**

Data on the use of antimicrobial agents in eighteen tertiary care hospitals were collected on March 20th 2002.

**Results:**

One or more antimicrobials were ordered in 2900 (30.6 %)of 9471 hospitalized patients. The reasons of hospitalization of the patients receiving antimicrobials were medical treatment (42.5 %), elective surgery (39.6 %), treatment of infectious disease (17.1 %) and emergent surgical procedures (10.4 %). The highest consumption frequencies were found in surgical (81.6 %) and medical (55.2 %) intensive care units. The 48.8 % of antimicrobials were given for treatment and 44.2 % for prophylactic use. The most common reasons for treatment were found as lower respiratory tract, urinary tract, surgical wound infections and febrile neutropenia. Antimicrobials were ordered empirically in 78.4 % of patients. The proven infection ratio was found as 30.7 %. The 56.4 % and 13.4 % of orders were evaluated as clinically and microbiologically appropriate respectively.

**Conclusion:**

These results suggest that antimicrobial prescription and empirical treatment ratios were high and inappropriate at inpatient groups.

## Introduction

Since antimicrobial chemotherapy was introduced in medical practice, there have been calls for its rational use. Appropriate antimicrobial treatment greatly improves the prognosis of infectious diseases. There has been a very significant reduction in morbidity and mortality associated with the use of antimicrobials since they were first introduced [1]. However, the overuse of antimicrobials may increase the risks of drug resistant pathogens, side effects and costs of medical care. The right agent at the right dose and dosing interval and right duration can achieve both a favorable clinical outcome and prevent the selection of resistance. It was reported that 20–50% of antimicrobial use in humans was questionable or inappropriate [1,2]. Accurate information about prescribing patterns in hospitals is valuable in improving the quality of antimicrobial prescriptions.

Only very limited data on the usage of antimicrobials in Turkey [1,3]. The over use of antimicrobials increases the risk of drug resistant pathogens, side affects and cost of medical care [4]. This multicenter study was planned as a point-prevalence study to evaluate antimicrobial prescription frequency and patterns in tertiary care hospitals in Turkey.

## Materials and methods

This prospective study was conducted in eighteen tertiary care hospitals from 14 different cities located in seven geographical regions of Turkey. These hospitals were representing approximately 30% of all tertiary care hospitals in Turkey. Data on the use of antimicrobial agents in these hospitals were collected by infectious diseases consultants on March 20^th ^2002. The same methodology was used for all hospitals. All patients who have received antimicrobials for any reason were included to this study. The data was included to the study within the first week of the study day, if there was a delay in the recording of data for any reason such as unavailable culture results which the specimen for this culture was collected before or on March 20^th ^2002.

In the study, total bed capacities of hospitals, number of hospitalized patients, the type and number of antimicrobial prescriptions, the main diagnosis which the prescription was made, clinical and microbiological evidences for treatment were recorded. The presence of an infectious disease was diagnosed according to signs and symptoms, non-microbiological and microbiological laboratory findings and defined as proven infection.

For patients receiving antimicrobials, demographic data, reason for admission and hospitalization, results of microbiological samples, name and dosage of antimicrobials and indication for antimicrobials were recorded on special forms. The antimicrobial regimes were evaluated according to choice, combination, duration and dose of the antimicrobials.

Hospital and patients details were recorded in two different forms. The first one was for the hospital details such as the name of the hospital, total bed capacity, departments and their bed capacities, total number of hospitalized patients of the hospital and of each department on the study day. The second form was used for recording data of patients which were receiving an antimicrobial agent on the study day. All of the records were collected and evaluated by the principal investigators in the study center. All of the data was transferred to the computer using a file designed by Dr. Ozgunes with Microsoft Access.

The antimicrobial prescription ratio, hospitalization reason of antimicrobial receiving patients and combination therapies were evaluated. Antimicrobial prescriptions were globally considered inappropriate if any of the assessed criteria appeared unacceptable, according to indication or antimicrobial choice, dosage errors, and duration of treatment. Appropriateness of antimicrobial prescriptions was evaluated according to the clinical and laboratory findings on the beginning of the therapy.

Statistical analyses were made by chi square test.

## Results

Eighteen tertiary care hospitals from 14 different cities of Turkey included to the study. 9471 hospitalized patients were evaluated. One or more antimicrobials were ordered in 2900(30.6%) of 9471 patients. In the antimicrobial receiving group 1232 (42.5 %) patients were hospitalized for medical treatment, 1147 (39.6%) for elective surgery, 497(17.1%) for infectious diseases, 303 (10.4 %) for emergent surgery and 73(2.5%) for other reasons. There were more than one hospitalization reasons for some patients (Table 1).

**Table 1:** Hospitalized, antimicrobial receiving patients and hospitalization reasons of antimicrobial prescribed patients in 18 centers.

	n	%
Hospitalized patients	9471	100

Antimicrobial receiving patients	2900	30.6

Hospitalization reason		

Medical treatment	1231	42.5
Elective surgery	1147	39.6
Treatment of an infectious diseases	497	17.1
Emergent surgery	303	10.4
Other reasons	73	2.5

The highest antimicrobial consumption ratios were found in intensive care units (ICU) (Surgical ICU 81%, medical ICU 52.5%). Antimicrobial consumption frequencies according to departments (surgical/medical) were shown in table 2.

**Table 2:** The distribution of antimicrobial prescribed patients to hospitalized patients.

	Antimicrobial prescribed	Total hospitalized	Percentage of antimicrobial prescribed
Surgical Clinics	1414	4172	33.9%*
Medical Clinics	1138	4529	25.1%*
Surgical ICU	107	132	81%*
Medical ICU	83	158	52.5%*
Total	2900**	9471	30.6%*

ICU: Intensive care unit.

*The ratios were found statistically different (x^2 ^= 119 SD = 2, P < 0.001).

**The wards of 158 patients were not reported.

The indications of antimicrobial therapy were also evaluated. The 48.8 % of antimicrobials were given for treatment of an infectious disease and 44.2 % for surgical antimicrobial prophylaxis. It wasn't found any reason for antimicrobial prescriptions in 204 (7 %) patients' records. More than one reason was reported for some patients.

Antimicrobial prescriptions were made empirically in 2275 (78.4 %) of patients and according to microbiological data in 334 (11.5%).

The proven infection ratio was found as 30.7 % in 2900 patients and 57.15% (807 of 1412) in treatment group. The antimicrobial prescriptions were evaluated by the investigator if or not they were appropriate to clinical and microbiological data. The 56.4 % and 13.4 % of orders were evaluated as clinically and microbiologically appropriate respectively in 2900 patients. In patients receiving prophylactic antimicrobials 671(52.46%) of 1279 prescription were evaluated as appropriate (Table 3). The 61.54% (869 of 1412) prescriptions were evaluated as clinically appropriate in patients receiving antimicrobials for treatment (Table 3). There was not any microbiological data in 986 (69.83%) patients in this group. The microbiologically appropriate prescription ratio was found 84.04% in 326 patients with microbiological data. The appropriate and inappropriate prescription in treatment group was given in table 3.

**Table 3:** The appropriate prescription in patients receiving prophylactic antimicrobials and the proven infection, clinically and microbiologically appropriate treatment ratios in patients that were treated for an infection.

		**n**	**%**
**Prophylactic antimicrobial use**	Appropriate	671	52.46*
	Inappropriate	423	33.07*
	Not reported	185	14.46
	TOTAL	1279	

**Clinically**	Proven infection	807	57.15
	Appropriate	869	61.54**
	Inappropriate	364	25.77**
	Not reported	179	12.67
	TOTAL	1412	100
**Microbiologically**	Appropriate	274	84.04***
	Inappropriate	52	15.95***
	Not reported	88	6.23
	TOTAL	414****	100
	No microbiological data	986	69.83

*t = 9.91, SD = 1092, p < 0.00

**t = 19.16, SD = 1231, p < 0.001

*** t = 15.79, SD = 324, p < 0.001

****The microbiological data was not available for all patients.

The combination therapy ratio was found as 33%. 50 patients including tuberculosis cases were receiving more than three antimicrobials. 25 patients were receiving combination therapy because of tuberculosis. 453 (15.6 %) of patients were receiving three antimicrobials and 428 of them (14.7%) were non-tuberculosis patients.

The most common prescribed antibiotics were cefazolin, ampicillin-sulbactam, ceftriaxone, ciprofloxacin, amikacin, gentamicin, ornidazole, cefuroxime, meropenem and vancomycin. The prescription ratios of antibiotic groups were given in table 4.

**Table 4:** The most common prescribed antibiotic groups in hospitalized patients and the most common used antibiotics in combinations.

**Antibiotic group**	**Prescription %**	**Combination %**
Penicillines	**23.6**	**18.8**
1.Generation Cephalosporins	**14.6**	**7.1**
2.Generation Cephalosporins	**5.3**	**0.0**
3.Generation Cephalosporins	**23.7**	**21.1**
4.Generation Cephalosporin	**4.2**	**4.5**
Aminoglycosides (Excluding streptomycin)	**17.2**	**30.8**
Carbapenems	**6.5**	**10.9**
Glycopolypeptides	**4.8**	**13.1**
Ornidazole-Metronidazole-Clindamycin	**9.9**	**18.2**
Quinolones	**14.4**	**11.9**
Macrolides	**3.0**	**4.7**
Tetracyclines	**0.7**	**1.2**
Antifungal agents	**3.4**	**4.3**

The most common used antibiotics in combinations were aminoglycosides (30.8%), 3^rd ^generation cephalosporins (21.1%), penicillins (18,8%), ornidazole-metronidazol-clindamycin (18.2%), glicopolypeptides (13.1%), quinolones (11.9%) and carbapenems (10.9%) (Table 4). The 88.5% of combined aminoglycosides were used in combination with beta-lactams and glycopolypeptides. There were 15 combinations of sulbactam-ampicillin with clindamycin, ornidazole or metronidazol. We determined that 67.44 % of the patients were in official health insurance systems and 19.7 % of them were in official social assistance system.

## Discussion

Although the principles of rational antimicrobial usage have been well defined for many years, inappropriate use of antimicrobials remains wide spread. The cost, adverse effects and development of resistance are main problems in wide spread usage of antimicrobials. The emergence and spread of drug resistant pathogens have already become a very serious problem internationally. It was reported that 14% and 43% of all courses of antimicrobial chemotherapy were deemed unnecessary because there was no evidence of infection [2,5,6].

In this study, antimicrobial prescription frequency was found as 30.6% in hospitalized patients. The antibiotic prescription frequency was reported as 77.8% from a university hospital in China, and as 65% from a pediatric teaching in Costa Rica [6,7]. Empirical antimicrobial prescription and combination antimicrobial treatment ratios were high (78.4%, 33%) in the study group also. The problem is more serious in ICU and surgical departments than medical departments. The antimicrobial prescription ratios were higher in ICU's (81% of surgical ICU, 52.5% of medical ICU) than other departments of hospitals (P < 0.001). It was reported that the 58.0% of surgical ICU patients in a university hospital from Germany were receiving antibiotics [8]. The antibiotic prescription frequency was reported as 6.55 and 14.4% from two different pediatric ICUs from Israel [9]. The proven infection ratio was found as 30.7% in the appropriate antimicrobial treatment given group and 57.9% in the inappropriate antimicrobial treatment group. The results of the study showed that inappropriate antimicrobial prescription was an obvious problem in the study hospitals of Turkey. More than 40 % of antimicrobial prescriptions were made without a proven infection. Inappropriate antimicrobial usage is a worldwide problem. 40% of antibiotic prescriptions were reported that had no record of justification and 55% of prescriptions had no indication of planned duration of therapy [7].

The 44% of antimicrobial prescriptions were made for surgical prophylaxis and 52.4% of them were appropriate. This group was seemed to be increasing the inappropriate prescription ratios because of the long duration usage and wrong selection of antimicrobials. Hu et al reported that 30% of hospitalized patients were receiving perioperative antibiotics and 20% of them received antibiotics before or during operation and 80% of them after operation. The duration of perioperative antibiotic prophylaxis was less than or equal to seven days in 42.7% of patients, 8–13 days in 31%, and 14 days or more in 26.3% [6]. In another study reported by Bailly et al, the rate of compliant prescription for surgical prophylaxis was 41.7% [10].

Also the combination therapy ratios were found as high as 33% of total antimicrobial prescribed patients. It can be thought that there is a relation between high empirical antimicrobial treatment and high combination therapy ratios. The limited microbiological evidence for the diagnosis of infection can be thought as another reason for high ratios of empirical and combination therapies because of the microbiologically appropriate and inappropriate usage ratios were found as 84.04% and 15.95% respectively in the treatment group. These results suggest that a multidisciplinary antimicrobial management system is required in hospitals because of the high proportion of empirically treatment and inappropriate use of antimicrobials. The system must have legal support and the antimicrobial control teams must be include the departments of infectious diseases, microbiology, pharmacy, and infection control [1]. Also there is need good microbiological support for clinicians to increase the appropriate prescription rate. Local and practical antimicrobial treatment guidelines for clinicians and continuous education programs may decrease the inappropriate, empirical and combination therapy ratios.

The cost of antimicrobials is another serious problem for insurance systems in Turkey. The anti-infective drugs are the most used drugs (22% of all drugs) in our country. The annually antimicrobial and total drug cost for per person was calculated as $8,4 and $38 in Turkey [11].

In conclusion, this point-prevalence study revealed that more than 50% of patients received inappropriate antimicrobial prescriptions. We thought that only restricted prescription procedures are not enough for the reduction of inappropriate antimicrobial rates. A general antimicrobial treatment program must include education, guidelines, restricted usage, control of the hospital pharmacies and automatic discontinuation by the hospital pharmacies.
